# The Challenges of Local Intra-Articular Therapy

**DOI:** 10.3390/medicina60111819

**Published:** 2024-11-05

**Authors:** Gailute Kirdaite, Jaroslav Denkovskij, Diana Mieliauskaite, Jolita Pachaleva, Eiva Bernotiene

**Affiliations:** 1 Department of Personalised Medicine, State Research Institute Centre for Innovative Medicine, LT-08406 Vilnius, Lithuania; 2 Department of Regenerative Medicine, State Research Institute Centre for Innovative Medicine, LT-08406 Vilnius, Lithuaniaeiva.bernotiene@imcentras.lt (E.B.); 3 Faculty of Fundamental Sciences, Vilnius Gediminas Technical University, VilniusTech, Sauletekio al. 11, LT-10223 Vilnius, Lithuania

**Keywords:** monoarthritis, inflammation, synovectomy, local treatment, fibroblast like synoviocytes, non-coding ribonucleic acids (RNAs)

## Abstract

Fibroblast-like synoviocytes (FLSs) are among the main disease-driving players in most cases of monoarthritis (MonoA), oligoarthritis, and polyarthritis. In this review, we look at the characteristics and therapeutic challenges at the onset of arthritis and during follow-up management. In some cases, these forms of arthritis develop into autoimmune polyarthritis, such as rheumatoid arthritis (RA), whereas local eradication of the RA synovium could still be combined with systemic treatment using immunosuppressive agents. Currently, the outcomes of local synovectomies are well studied; however, there is still a lack of a comprehensive analysis of current local intra-articular treatments highlighting their advantages and disadvantages. Therefore, the aim of this study is to review local intra-articular therapy strategies. According to publications from the last decade on clinical studies focused on intra-articular treatment with anti-inflammatory molecules, a range of novel slow-acting forms of steroidal drugs for the local treatment of synovitis have been investigated. As pain is an essential symptom, caused by both inflammation and cartilage damage, various molecules acting on pain receptors are being investigated in clinical trials as potential targets for local intra-articular treatment. We also overview the new targets for local treatment, including surface markers and intracellular proteins, non-coding ribonucleic acids (RNAs), etc.

## 1. Introduction

The European League Against Rheumatism (EULAR) has launched a Europe-wide campaign, “Don’t delay, connect today”, to raise awareness of the major public health concerns of rheumatic and musculoskeletal diseases (RMDs). Guidelines from the Osteoarthritis Research Society International (OARSI) and the Food and Drug Administration (FDA), as well as recommendations from the EULAR, emphasize the importance of early anti-inflammatory therapy (EIT) and the treat-to-target strategy (T2T) [1,2,3]. According to EULAR recommendations, a rheumatologist should see the patient within 6 weeks of the onset of arthritis and decide on a treatment strategy. The “window of opportunity” is usually up to three months, which is a crucial timeframe to choose an effective treatment strategy to stop the progression of the disease in its early stages [4,5].

Therefore, the management of inflammatory arthritis, especially monoarthritis (MonoA), before it develops into oligoarthritis and polyarthritis remains a challenge. The duration of acute MonoA is two to four weeks. Making an accurate diagnosis in such a short period is only sometimes possible. In most cases, acute MonoA is caused by a variety of causes ranging from benign to life-threatening. Thus, the onset of inflammatory arthritis (IA) may be MonoA, which needs to be carefully differentiated from diseases with different pathogenesis, such as gout (urine-type crystals), traumatic arthritis (trauma), infectious arthritis (virus or bacteria), paraneoplastic arthritis (cancer), and others [6,7,8]. It should be noted that several cases of MonoA have also been described since the beginning of 2021 after COVID-19 [9].

The PubMed scientific literature data on this topic could be more coherent, so we decided to review these issues. Thus, it remains to resolve and understand the main problem of MonoA as a choice of intervention strategy for synovial local inflammation. According to the literature, about 50% of MonoA cases resolve spontaneously; the rest develop into oligo- or polyarticular disease, but a significant proportion remains as persistent inflammatory MonoA. We attempt to analyze the data based on a basic pathogenetic message that FLSs are the key players in the majority of MonoA cases. As FLSs are key targets for local therapy, we focused on new targets related to surface markers and intracellular proteins, non-coding ribonucleic acids (RNAs), signaling, etc. Therefore, there is an urgent need to suppress or eradicate local inflammation, from which aggressive fibroblast-like synoviocytes (FLSs) are activated to induce cartilage degradation and may trigger a systemic autoimmune response [10,11], including rheumatoid arthritis (RA). Synovial inflammation has recently been suggested as a target for the treatment of osteoarthritis (OA) as well [12,13]. Thus, the synovitis-related phenotype or endotype of OA is an important factor in designing more effective disease-modifying interventions in these cases [14]. There is very little knowledge on the incidence and prevalence of early IA in primary care settings. Therefore, in most studies, the prevalence of early undifferentiated arthritis is around 30% [15,16,17].

Today, the scientific data on local synovectomy are well studied, one by one, but it is necessary to summarize the comparative data on this treatment modality. These challenges concern the scientific, medical, and pharmaceutical communities as they seek to establish a strong link between new intra-articular (i.a.) treatments. Thus, we analyzed local treatments from the US National Library of Medicine (clinicaltrials.gov, accessed on 1 January 2023) database with the following criteria: 2013–2023 years completed trials with results publications, adult population, and intra-articular arthritis treatment (in particular anti-inflammatory effects on synovium).

Therefore, in this paper, we will analyze the long-term monitoring results of MonoA and explore the local treatment strategies available today.

## 2. Materials and Methods

We tried to analyze the data based on the pathogenesis basic knowledge that FLSs are key players in most cases of arthritis (see graphical abstract). In the 1st step, the PubMed database was searched for the keyword ‘monoarthritis’ only. Monoarthritis was represented by 353 case reports, but these cases were not included in the pooled data.

Therefore, in the 2nd step, we had to solve and understand the main problem of the choice of the intervention strategy for local inflammation of the synovium, so we used different combinations of terms for local treatment approaches in the PubMed database search: ‘monoarthritis’, ‘local treatment’, ‘intra-articular treatment’, ‘synovectomy’, and ‘synovitis treatment’. On the basis of our analysis of these clinically evaluated synovectomies, we concluded that there is still a lack of data on selective local treatment.

In the 3rd step, we analyzed 53 contemporary local treatments from the US National Library of Medicine (clinicaltrials.gov) database according to the following criteria: completed trials with results, 10-year follow-up period, adult population, and intra-articular arthritis treatment. In the clinical studies, we searched for new intra-articular approaches, especially for OA patients with inflammatory phenotype where FLSs are less aggressive.

In the 4th step, we asked about new advances in the inhibition of aggressive FLS suppression, using an RA example. As FLSs are key targets for local therapy, we focused on new targets related to surface markers and intracellular proteins, non-coding ribonucleic acids (RNAs), signaling, etc.

## 3. Local Treatment of Arthritis

### 3.1. Monoarthritis: Treatment Challenges

**Figure 1:**
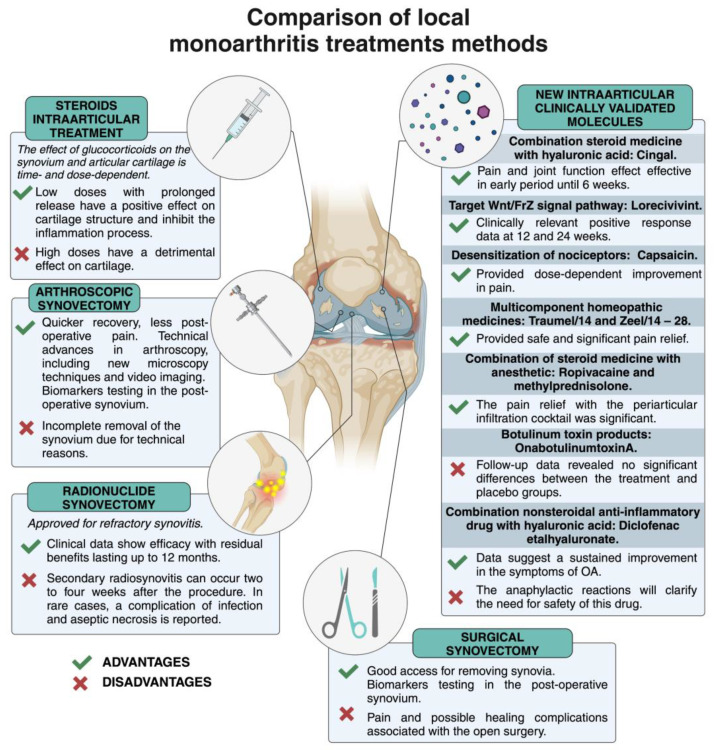
Advantages and disadvantages of local monoarthritis treatments. Created with BioRender.com (accessed on 4 July 2024).

The experience with MonoA shows that local treatment of joint synovitis is feasible, especially when large joints are affected (Figure 1).

In general, each of the local procedures used in clinical practice has its own advantages and disadvantages, and open and arthroscopic surgical interventions remove inflamed tissue while providing the opportunity to study biomarkers [18,19,20]. Currently, needle ultrasound and arthroscopy biopsy are used to assess synovitis [21,22], but new contrast-enhanced magnetic resonance imaging (MRI) techniques are better able as a reference method to characterize the synovium and cartilage [23]. A decrease in orthopedic joints is linked to the development of more conventional new systemic biologics, medications, etc. [24,25].

The EULAR recommendations on intra-articular therapy for different inflammatory arthritis should be considered [26]. The effect of corticosteroids on articular cartilage is time- and dose-dependent: a low dose has a beneficial effect, whereas a high dose has a detrimental effect on cartilage degradation [27]. There is a diverse scientific debate on the predictive factors of response to intra-articular steroid injections in knee osteoarthritis: degree of synovitis, site of discharge, and cartilage protection [28]. Intra-articular injections of glucocorticoids are probably the most common and widely used for local inhibition of synovial inflammation. This EULAR Guideline describes the basic principles for the use of i.a. steroid injections for knee pain relief in combination with release or for RA in cases where there is a need to adjust the disease-modifying antirheumatic drugs DMARDs therapy in one or more remaining active joints [26]. As previously described, in the MonoA, steroid therapy is the first choice for suppressing local inflammation; this treatment modality is good at suppressing synovitis and symptoms. In cases where steroids are injected close to the synovium (e.g., into the fat pad of the knee joint), the anti-inflammatory efficacy may be very similar to the same precise needle placement into the synovial cavity due to the distribution of the steroid through the tissues surrounding the joint [29]. Synovial inflammation has recently been proposed as an essential target for the treatment of OA [12]. Today, the FDA has approved low-dose, slow-release drugs for the treatment of OA local synovitis [30].

### 3.2. Recent Developments of Intra-Articular Medication

We analyzed and summarized 10 years of clinical studies in Table 1. Thus, novel molecules have been described as clinically validated i.a. therapies for synovitis treatment strategies. Joint damage due to synovial inflammation and cartilage breakdown are two of the factors that cause pain. Therefore, analgetic treatment is also very essential for local treatment. Brief comments on the solutions to these problems are given below.

Summary: Thus, today, new steroid formats are approved for the local treatment of OA knees, hips, and shoulder joints with a slow-acting drug that has no detrimental effect on the cartilage or other aspects of the joint structure; the pain relief is dose-dependent. The 3b clinical study with a flexible dosing schedule repeated twice according to patient response was well tolerated and effective until 52 weeks [33]. Other studies in which steroids were administered slowly showed encouraging results in terms of pain relief and improvement in physical function at around 24 weeks [30,31,32,34,35,36,37]. Another important aspect is the combination of steroids with HA, which has been evaluated and found to have a beneficial anti-inflammatory effect in combination with viscosupplementation [38]; this combination is effective against pain in the early phase of the disease for up to 6 weeks. WNT inhibitors are being investigated and this drug has a positive effect on cartilage degeneration by affecting chondrocyte differentiation and inhibition of osteoblasts and synovial cells [39,40]. Meanwhile, molecules, involved in the long-term desensitization of nociceptors associated with calcium influx into nociceptive nerve endings show a beneficial pain inhibitory effect [42]. The regulation of the nociceptive response with botulinum toxin has not shown a beneficial effect on synovium pain [45]. Various combinations (steroids with anesthetics or NSAIDs with HA) are currently being clinically validated, and these agents have shown pain suppression [44,46,47]. Attention must be paid to nonsteroidal anti-inflammatory drugs in combination with HA regarding anaphylactic reactions [47]. Consequently, multicomponent homeopathic medicines are also used in the clinical practice for pain relief [43]. The effects of all of these drugs are dose-dependent, with duration ranging from 12 weeks to 52 weeks. The summarized data are represented in Figure S1. Local treatment of early synovitis with targeted intra-articular suppression is essential, but at the same time, the per os treatment must be chosen to select an effective treatment strategy and to halt disease progression in the early stages [4,5]. MonoA data for local treatment, especially intra-articular treatment, have revealed that key target molecules are involved in OA. In the clinical studies described above, we analyzed novel molecules approved for intra-articular therapy, particularly targeting OA patients with an inflammatory phenotype where FLSs are less aggressive. Another approach is the investigation of new targets in inflammatory tissues after synovectomy in pre-clinical studies. As fibroblast-like synoviocytes are one of the targets of local treatment, we focused on these synovitis-promoting players. The behavior of FLS in RA is an excellent example for exploring new targets related to surface markers and intracellular proteins of these cells, non-coding RNAs, signal transduction, etc.

### 3.3. Fibroblast-like Synoviocytes as Potential Targets for Early Local Therapy

**Figure 2:**
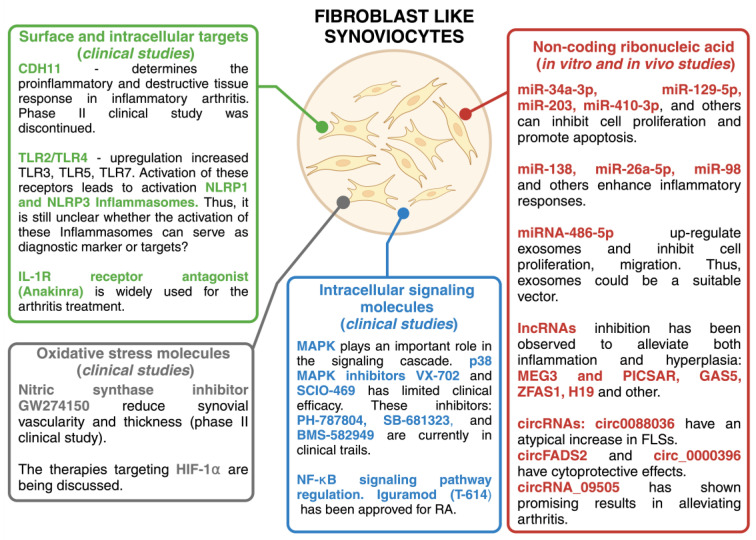
Potential therapeutic targets related to FLSs. Created with BioRender.com.

In RA, fibroblast-like synoviocytes have intrinsic pathogenic properties and actively contribute to the disease process. They proliferate and promote joint destruction by stimulating inflammation. However, the reasons why they turn from beneficial to harmful in RA remain to be fully understood. Studies have shown that persistent inflammation can induce molecular changes in FLSs that transform them from passive responders to inflammation to active aggressors [48,49]. RA FLSs have specific characteristics that distinguish them from healthy FLSs. These characteristics remain unchanged even when RA FLSs are isolated from an environment rich in inflammatory cytokines [11,49]. Potential therapeutic strategies could target FLS surfaces and intracellular proteins, FLS metabolism, and signaling pathways that increase FLS invasive and migratory potential, non-coding ribonucleic acid, oxidative stress molecules, etc. Current therapeutic approaches focus on modifying the immune response, specifically by targeting pro-inflammatory cytokines, B cells, or T cells [50]. Some of these drugs may also influence the invasive behavior of FLSs, especially those that inhibit cytokines or signal transduction pathways [51,52]. Although these drugs can reduce the level of FLS activity in RA, they may not always be effective, which is why patients still suffer from this disease. In such cases, the use of a combination of alternative or complementary therapies may be useful to manage the disease effectively. Potential therapeutic targets related to FLS are described in Figure 2 and the following sections.

#### 3.3.1. Cell Surface Targets

The discovery of different FLS phenotypes that contribute to synovitis leads to the possibility of targeting cells based on their surface phenotype. This implies the identification of pathogenic cell-specific surface proteins and the development of innovative treatment strategies targeting specific RA FLS phenotypes. RA FLSs lineages are characterized by the expression of specific markers such as CD10, CD34, CD55, CD90, CD248, and podoplanin (PDPN) [53,54,55,56]. These surface markers characterize RA FLS subgroups with significant functional differences. For example, previous studies have shown that cadherin 11 (CDH11) significantly contributes to homotypic FLS aggregation in in vitro and in vivo models [57,58]. CDH11 has attracted considerable attention as a potential marker of RA and has been widely regarded as a promising target. Nevertheless, a clinical trial investigating the efficacy of a monoclonal antibody directed against CDH11 in a phase II study was discontinued due to low efficacy.

Other potential markers and targets of RA include Toll-like receptors (TLRs) and inflammasomes. Pattern recognition receptors (PRRs) such as TLRs recognize pathogen-associated molecular patterns (PAMPs) or damage-associated molecular patterns (DAMPs). If regulatory mechanisms fail, TLR activation can trigger local inflammation and contribute to inflammatory or autoimmune diseases. Necrotic cells in inflamed joints may be a source of endogenous ligands for these receptors. Heat shock proteins and low molecular weight hyaluronan were originally thought to activate TLR2/TLR4 heterodimers directly, but pure ligands do not effectively activate these receptors [59]. However, citrullination of endogenous ligands such as fibrinogen and histones can stimulate the TLR4-mediated pathway [60,61]. The involvement of the TLR4-mediated pathway in the development of RA is suggested by the significant inhibition of monocyte activation observed in these patients with ACPA when treated with anti-TLR4 antibodies [62]. TLR4 can increase the production of pro-inflammatory cytokines and chemokines, such as IL-6 and IL-17, by binding to exogenous ligands, such as peptidoglycan, in the FLS of RA patients and in peripheral blood mononuclear cells (PBMCs), leading to inflammation and degeneration of cartilage [63]. In addition to the up-regulation of TLR2 and TLR4, an increased expression of TLR3, TLR5, and TLR7 has been observed in FLSs from RA patients compared to individuals with OA or without inflammatory diseases [64,65,66,67]. Activation of these receptors leads to enhanced local inflammatory responses, including the formation of nucleotide-binding oligomerization domain (NOD)-like receptor family pyrin domain-rich receptor 1 (NLRP1) and NLRP3 inflammasomes [68].

Inflammasomes are multicellular protein complexes that activate the release of pro-inflammatory cytokines such as interleukin-1β (IL-1β) in response to cellular stress or infection. Activation of the NLRP3 inflammasome contributes to the development of autoimmune diseases such as ankylosing spondylitis, systemic sclerosis, systemic lupus erythematosus, and RA [69,70]. Two signals are required to activate the NLRP3 inflammasome. The first signal is transduced via membrane receptors such as TLRs. Meanwhile, the second signal is linked to stimuli such as changes in ATP, K^+^ and Ca^2+^ levels, lysosomal destabilization, mitochondrial dysfunction, reactive oxygen species, and uric acid crystals [71,72,73]. Targeting TLRs and inflammasomes is promising for modulating the immune response and possibly treating inflammatory disorders. However, it is still unclear whether the activation of these inflammasomes can serve as diagnostic markers to differentiate undifferentiated early inflammatory arthritis into specific diseases such as RA.

The question is whether these different populations are definitive subsets with a consistent phenotype or whether the FLS phenotype is flexible and microenvironment-dependent, leading to differences in the relative prevalence of the various putative phenotypes.

#### 3.3.2. Intracellular Signaling Molecules

Various cell signaling molecules have been investigated as potential biomarkers of RA, which can help diagnose, monitor, and predict the disease’s progression.

There are several strategies to combat FLSs, including inhibition of components of the mitogen-activated protein kinase (MAPK) cascade, inhibition of kinases that activate c-Jun N-terminal kinase (JNK), and blocking the nuclear factor-κB (NFκB) pathway [74].

MAPK regulates the production of pro-inflammatory cytokines and plays an important role in the signaling cascade downstream of interleukin (IL)-1, IL-17, and tumor necrosis factor (TNF)-α receptors [75,76]. Activation of MAPK family members occurs primarily in synovial tissues. Their activation is important for the production of pro-inflammatory cytokines such as TNF, IL-6, and IL-1. p38 kinase is a potential target for the treatment of RA, but clinical trials have yet to identify effective inhibitors. The results show that the p38 MAPK inhibitor VX-702 has limited clinical efficacy and is accompanied by transient inhibition of inflammatory biomarkers [77]. However, it may not offer a significant and permanent inhibitory impact on the chronic inflammation observed in RA. For instance, the clinical trial, including SCIO-469, an orally administered inhibitor of p38-α MAPK, did not show greater efficacy than a placebo in individuals diagnosed with rheumatoid arthritis [78]. PH-797804, SB-681323, and BMS-582949 are p38 inhibitors currently in clinical trials [74]. As an alternative approach, higher members of the MAPK cascade, such as MKK3, MKK6, and MAP3K5, have been investigated as potential targets in pre-clinical models [79].

Targeting JNK in this pathway has shown encouraging results in alleviating the clinical symptoms of RA. JNK1 plays a crucial role in maintaining and promoting inflammation in the synovium [80]. It is expressed in FLS and macrophage-like synoviocytes (MLS), and targeting JNK1 with blocking agents can reduce its endogenous expression in both synovial cell types [81,82]. For example, the use of the JNK inhibitor SP600125 has decreased c-Jun transcription and enhanced the accumulation of phospho-Jun, thereby attenuating the inflammatory response. In addition, the JNK inhibitor AS601245 can relieve symptoms of collagen-induced arthritis rat model (CIA) rats [83].

Current pharmacological drugs used for treatment therapy specifically target or interact with the NF-κB signaling pathway. The NF-κB signaling pathway regulates a variety of cellular processes, including inflammation, immune response, and cell survival [74,84]. These drugs act by modulating NF-κB activity, inhibiting its activation or blocking downstream signaling events. For example, methotrexate (MTX) is an effective drug for the treatment of RA and affects TNF-α levels in early RA patients via the NF-κB pathway [85]. Prednisolone, a synthetic glucocorticoid, inhibits the transcription of inflammatory genes via the NF-κB signaling pathway and is clinically used to reduce RA inflammation [86].

In addition, new drugs are currently being investigated and developed to reduce the activity of aggressive FLS by modulating the NF-κB signaling pathway. Iguramod (T-614) is a new disease-modifying anti-rheumatic drug that inhibits NF-κB activation and is approved for the treatment of RA in Japan and China [87]. Denosumab inhibits NF-κB ligand–receptor activator and can partially restore bone erosions in RA patients. The combination of Denosumab with DMARDs may be considered in RA patients with progressive bone erosions. Previous studies have demonstrated the efficacy of an antagonist targeting cysteinyl leukotriene receptor 1 (CysLT1) in inhibiting NF-kB pathway activation as well as interleukin-6 (IL-6) and interleukin-8 (IL-8) secretion in FLS [88]. The results of this study suggest that modulation of CysLT1 and leukotriene B4 (LTB4) receptors may be an effective therapeutic strategy to reduce inflammation and slow the progression of RA patients [89]. However, further studies are needed to confirm their efficacy and to investigate their clinical application. The integration of multiple biomarkers and the use of advanced technologies may increase their diagnostic and prognostic value in the future [10,90,91].

#### 3.3.3. Non-Coding Ribonucleic Acids

Non-coding ribonucleic acids (ncRNAs) have emerged as potential biomarkers for rheumatoid arthritis (RA). ncRNAs are RNA molecules that do not encode proteins but play important regulatory roles in gene expression and cellular processes, including signaling pathways. ncRNAs comprise a diverse group of molecules, including microRNAs (miRNAs), long non-coding RNAs (lncRNAs), and circular RNAs (circRNAs).

The level of miRNAs can influence the secretion of inflammatory cytokines or metalloproteinases (MMPs), which in turn can impact the progression of RA [92]. Certain miRNAs have been found to inhibit cell proliferation and promote apoptosis [93], while others have been shown to contribute to the inflammatory environment, possibly leading to tissue damage [94,95]. Animal and cell culture experiments have shown promising results when certain miRNAs alleviate or enhance RA symptoms. For example, when overexpressed, miR-34a-3p [96], miR-129-5p [97], miR-203 [98], miR-410-3p [99], and others can inhibit cell proliferation and promote apoptosis by targeting different proteins. On the other hand, miR-138 [100], miR-26a-5p [94], miR-98 [101], and similar miRNAs enhance inflammatory responses. In addition, miRNA-486-5p up-regulation in exosomes has been found to inhibit cell proliferation and migration, suggesting that exosomes could be a suitable vector for the therapeutic delivery of miRNA-486-5p [102]. Long non-coding RNAs (lncRNAs) over 200 nucleotides in length are widely expressed in many human tissues and can be a diagnostic tool for RA [103]. However, like miRNAs, they may have dual effects. Some lncRNAs have anti-inflammatory properties, while others can enhance inflammatory reactions. For example, inhibition of specific long non-coding RNAs (lncRNAs) has been observed to alleviate both inflammation and hyperplasia. The involvement of non-coding RNAs (ncRNA) FER1L4 and MEG3 in RA has been demonstrated in [104,105]. MEG3 upregulation has an inflammation-suppressive effect by modulating the AKT/mTOR signaling cascade [106]. PICSAR, an additional (ncRNA), influences several cellular processes, including cell proliferation, migration, invasion, and the synthesis of IL-6, IL-8, and MMP-3. It exerts this influence by interacting with miR-4701-5p [107]. The role of the miR-222-3p/Sirt1 axis is central to the action of GAS5 in mitigating RA FSL proliferation, inflammation, and apoptosis [108]. Silencing of the lncRNA ZFAS1 may mitigate inflammation and hyperplasia by competitively binding to miR-296-5p and regulating MMP-15 expression in the context of an experimental arthritis model. Inhibition of ZFAS1 has been observed to alleviate both inflammation and hyperplasia. This effect is achieved by binding ZFAS1 to miR-296-5p, which subsequently regulates MMP-15 expression [109]. Furthermore, IncRNAs expressing lncRNA-H19 injected into the ankles of collagen-induced arthritis (CIA) mice ameliorate the progression by competing with miR-124a, which directly acts on CDK2 and MCP-1 [110].

Circular RNAs (circRNAs) are a recently discovered class of endogenous ncRNA molecules whose importance in regulating gene expression is increasingly recognized. CircRNAs have been found to be abnormally elevated in RA and to contribute to disease progression. These circRNAs have been identified as abnormally increased in RA and contribute to the advancement of the disease. These ncRNAs have great potential and promising targets for the treatment of RA. They have several functional properties, including RNA polymerase II elongation, regulation of RNA maturation, and protein localization [111]. Several studies have started to investigate the involvement of circRNAs in the pathogenesis of RA. As an example, the molecule circ0088036 has been observed to have an atypical increase in FLSs. This abnormality contributes to the progression of RA by acting as a molecular sponge for miR-140-3p, thereby augmenting the production of SIRT 1 [112]. Other circRNAs, circFADS2 and circ_0000396, have been shown to have cytoprotective effects against apoptosis and suppress cell proliferation [113]. Furthermore, it has been observed that circRNA_09505 plays a significant role in promoting the expression of AKT1 by regulating the IkBa/NF-kB signaling pathway in macrophages. Notably, the knockdown of circRNA_09505 has shown promising results in alleviating arthritis and inflammation in mice with collagen-induced arthritis (CIA) [114]. RNA plays a crucial role in the pathophysiology of rheumatoid arthritis (RA) and has great potential for diagnostic and therapeutic target treatment. Despite challenges related to identification and characterization, tissue specificity, standardization and reproducibility, functional characterization, validation, and clinical utility, ncRNA are promising potential biomarkers of RA due to their stability, detectability in various body fluids, and involvement in regulatory processes [115,116,117]. However, further studies are needed to fully understand the complex mechanisms underlying these different forms of RNA.

#### 3.3.4. Oxidative Stress Molecules

Oxidative stress is defined as a harmful condition characterized by an imbalance of oxidative molecules, such as reactive oxygen species (ROS), leading to an excess of prooxidants [118]. This imbalance can lead to disruption of redox signaling and molecular damage. Under physiological conditions, ROS are required to maintain the cellular redox status and play an important role in cell signaling pathways, differentiation, proliferation, growth, apoptosis, regulation of the cytoskeleton, and phagocytosis. However, when ROS levels exceed physiological levels, they can have detrimental effects on many cellular components, such as cell membranes, lipids, proteins, and nucleic acids [119,120]. In RA patients and animal models, there is a significant association between blood ROS levels and RA severity. Several studies have shown alteration in the expression of nitric oxide (NO) and inducible nitric oxide synthase (iNOS), which lead to impaired infiltration of T- and B-cells into the joints by interfering with their chemotaxis and adhesion [121,122]. Furthermore, promising results have been observed with NOS and iNOS inhibitors L-NAME and iNOS inhibitor GW274150 in reducing the inflammatory response and synovial thickness, offering the potential for targeted treatments [123,124]. In addition, patients with active RA are characterized by increased ROS levels and reduced antioxidant capacity, leading to increased levels of lipid peroxidation, which can be observed in synovial fluid and blood samples [125,126,127,128]. Based on previous studies, a positive correlation between lipid peroxidation biomarker malondialdehyde (MDA) and proinflammatory cytokines has been observed in the serum of individuals with RA [129]. Furthermore, studies have shown that reactive oxygen metabolites (ROM) are increased in blood samples from RA patients and positively correlate with disease activity [130]. In line with these findings, it has been observed that RA patients have reduced levels of antioxidants in serum and synovial fluid [131,132].

A link between oxidative damage to synovial tissue, mitochondrial dysfunction, and hypoxic status in arthritic joints has also been demonstrated. For example, inflammatory mediators and hypoxic conditions can impair the mitochondrial state of synovial cells, leading to metabolic shifts and increased mutation rates. Furthermore, oxidative stress can alter energy metabolism, increase ROS production, and raise mitochondrial mutagenesis, thereby contributing to inflammatory processes and impaired angiogenesis in RA patients. The metabolic disparity between healthy synovial tissue and RA-affected synovial tissue particularly highlights the alterations in cellular metabolism known as the Warburg effect or aerobic glycolysis [133]. This phenomenon describes the overexpression of glycolytic enzymes in RA synovial tissue and its potential impact on inflammatory cytokines, cell proliferation, and disease activity [134,135]. In the context of RA, studies have shown that hypoxia-inducible factor (HIF)-1α is important in maintaining oxygen balance and regulating the expression of genes involved in angiogenesis and inflammation in the synovium. Various factors, including hypoxia, ROS, cytokines, hormones, and mechanical stress, influence HIF-1α activation. In terms of clinical relevance, the potential of therapies targeting HIF-1α or angiogenic factors as an alternative approach to the treatment of RA has been suggested [136,137]. Furthermore, studies on the Notch signaling pathway in the context of RA have revealed its role in regulating cellular processes and promoting inflammation. It has also been suggested that the Notch signaling pathway could be used as a pharmacological treatment for RA [138].

In general, local treatment of early synovitis with conventional glucocorticoids and synovectomy involves targeted suppression or eradication of aggressive fibroblast-like synoviocytes. In advanced RA, combining immunosuppressants with local FLS-targeted therapy can more effectively control disease activity.

## 4. Conclusions and Future Directions

In the course of MonoA, local synovitis treatment involves targeted inhibition or destruction of fibroblast-like synoviocytes. Today, several new intra-articular therapies have been approved in clinical studies, especially for osteoarthritis patients with an inflammatory endotype where FLSs are less aggressive. Currently, various slow-acting steroidal drugs for the local treatment of synovitis, which do not have a detrimental effect on cartilage, are gradually being marketed in clinics. Various combination therapies (steroids with HA, steroids with anesthetic, nonsteroidal anti-inflammatory drugs with HA, etc.) are currently approved in clinical practice and have proven to be effective in pain relief. Thus, personalized medicine initiatives involve the choice and decision of medicines based on the unique clinical characteristics or risk factors and biomarker expression of each patient [130]. A local approach to the elimination of synovial tissue inflammation will lead to the discovery of new local targets related to surface markers and intracellular proteins, non-coding RNAs, signaling molecules, and improvement in the treatment techniques and protocols.

## Figures and Tables

**Table 1:** New clinically tested treatment options for local synovial inflammation.

Clinical Studies Showing Positive Local Intra-Articular Anti-Inflammatory and Analgesic Effects		
STEROIDS		
1. Triamcinolone acetonide extended-release. Product name and description: FX006 (Zilreta), a microsphere-based extended-release triamcinolone acetonide.		
Phase	Design (*n* = patients), tested medication doses, follow-up duration.	Outcomes
Phase 2	Double-blind, multicenter study (228 patients); Dose FX006 (containing 10, 40, or 60 mg of triamcinolone acetonide) or 40 mg of immediate-release triamcinolone acetonide (TA). Follow-up 12 weeks.	FX006 40 mg dose provided pain relief from 2 to 12 weeks. This medication was significantly superior to TA at five to ten weeks. Adverse events were mild and similar in the treatment groups [31].
Phase 2b	306 participants were randomized (1:1:1) to receive single i.a. injections of FX006 32 mg (*n* = 104) or 16 mg (n = 102) or saline placebo (*n* = 100).	FX006 32 mg versus 16 mg produced a dose-dependent analgesic response, the treatment was well tolerated, and the safety profile was similar to that of saline placebo [32].
	Follow-up 12 weeks.	
Phase 3b	Single-arm, open-label study, participants (*n* = 208). All patients received the first (day 1) intra-articular triamcinolone acetonide extended-release (TA-ER) injection. The second-injection patients received a flexible (at either week 12, 16, 20, or 24) schedule, which depends on the first-injection response duration. Patients who received two injections were evaluated for 52 weeks.	TA-ER administration with a flexible dosing schedule repeated twice according to patient response was well tolerated, with no radiographic evidence of knee joint cartilage effects [33].
Phase 2	Randomized, multicenter, open-label, single-dose study, 30 patients with hip OA were randomly assigned 1:1 to receive single i.a. injections in the hip joint of TA-ER 32 mg or triamcinolone acetonide TA crystalline suspension (TAcs) 40 mg. Pharmacokinetic (PK) analysis of this medication concentration was performed up to day 85.	The systemic plasma concentration exposure was substantially lower for TA-ER versus TAcs during follow-up. Thus, TA-ER was generally well tolerated, with a safety profile comparable to that of Tacs [34].
Phase 2	Randomized, open-label, single-dose study 25 patients randomly received a single ultrasound-guided i.a. injection in the shoulder joint of TA-ER 32 mg or TAcs 40 mg. PK analysis of these medication concentration was performed up to day 85.	The pharmacokinetic results showed that TA-ER was well tolerated with the systemic safe profile compared to TAcs crystalline suspension [35].
Phase 2	Open-label study sequentially enrolled 81 patients. After SF aspiration, the i.a. injection of 32 mg FX006 or 40 mg TAcs was given to each patient at baseline and during follow-up visits (FX006: weeks 1–6–12–16–20; TAcs: week 6).	After injection of FX006 microspheres, the SF and plasma PK observations are consistent with slow release of TA relative to TAcs in patients with knee OA. The slow release of FX006 microspheres reduced systemic TA exposure relative to TAcs [36].
2. Liposome formulation of dexamethasone sodium phosphate. Product name and description: TLC599 is a water-soluble corticosteroid, dexamethasone sodium phosphate (DSP), produced using new BioSeizer^®^ technology, which encapsulates therapeutically active molecules in multilayer lipid membranes to ensure an extremely long drug release time.		
Phase 2a	Randomized, placebo-controlled, dose-finding study, *n* = 75 patients. Two single injections of TLC599 (12 mg and 18 mg DSP) and placebo (normal saline) were given, and pain relief was observed in clinical data up to week 24.	The 12 mg dose level of TLC599 showed effective pain relief and improvement in physical function. Treatment was safe and without side effects [37].
3. Combination steroid medicine with hyaluronic acid (HA). Product name and description: Triamcinolone hexacetonide with a commercial cross-linked HA viscosupplement (Cingal).		
	Multicenter, double-blind, saline-controlled clinical trial randomized 368 subjects. A single injection of Cingal (4 mL, 88 mg HA plus 18 mg triamcinolone hexacetonide (TH)), Monovisc (4 mL, 88 mg HA), or saline solution (4 mL, 0.9%). The pain score monitoring was assessed at 12 weeks and 26 weeks.	The pain and function were significantly better in Cingal group compared to saline groups. Cingal had a better effect in the early period, but after 6 weeks and beyond, the effect was similar compared to Monovisc alone [38].
WNT INHIBITORS		
4. Target Wnt/FrZ signal pathway. Product name and description: SM04690 (Lorecivivint (LOR)), which acts as an inhibitor of the Wnt signaling pathway, is thought to play an important role in cartilage degeneration by affecting the differentiation of chondrocytes, osteoblasts, and synovial cells.		
Phase 2b	Multicenter, randomized, double-blind, placebo-controlled trial, *n* = 695 patients. This study’s objective was to identify effective LOR doses: 0.03, 0.07, 0.15, or 0.23 mg. Follow-up 24 weeks.	The optimal dose was identified as 0.07 mg LOR [39].
	Post hoc analysis of this clinical trial identified the proportions of LOR responders.	Patient-reported outcomes (PROs) provide clinically relevant positive response data on measures of pain, function, and patient global assessment compared with placebo, with benefits that persisted at 24 weeks [40].
	Post hoc analysis of this clinical trial identified the proportions of LOR responders.	The PRO comparison of control groups, dry needle sham, and saline-based placebo injections for the treatment of knee OA showed equivalent patient-reported outcomes at all time points up to week 24, with no physiological impact [41].
Summary: The results of the clinical study suggested that the optimal dose for future studies was 0.07 mg LOR [39]. A post hoc analysis yielded clinically relevant positive response data at 12 and 24 weeks in participants with moderate and severe knee OA [40,41].		
PAIN MANAGEMENT		
5. Molecule induces desensitization of nociceptors. Product name and description: The Capsaicin (CNTX-4975) molecule is involved in the long-term desensitization of nociceptors associated with calcium influx into nociceptive nerve endings.		
Phase 2	Multicenter double-blind study, *n* = 172 patients. Randomization: placebo group, CNTX-4975 0.5 mg and 1.0 mg groups. Follow-up 24 weeks.	CNTX-4975 1.0 mg at 24 weeks significantly reduced OA knee pain; CNTX-4975 0.5 mg significantly reduced pain at 12 weeks, but no effect on pain effect was evident at 24 weeks. Adverse events were mild and similar in the treatment groups [42].
6. Multicomponent homeopathic medicines. Product name and description: Traumel/14 and Zeel/14–28 ingredients are of vegetable, mineral, and other organic origin, diluted to so-called “low strength” because they contain measurable molecular concentrations of potential active substances with anti-inflammatory, antioxidant, and chondroprotective properties.		
	The double-blind, multicenter, randomized, saline-controlled trial, *n* = 232 patients. The investigational medicinal product is 4.2 mL (2.0 mL for Zeel/14 and 2.2 mL for Traumel/14 on days 1, 8, and 15 of treatment). Follow-up 29, 71, 85 days.	In this study, intra-articular Traumel14/Zeel14 provided safe and significant pain relief [43].
7. Combination of steroid medicine with anesthetic. Product name and description: Ropivacaine and methylprednisolone were infiltrated with the periarticular infiltration of this cocktail for the reduction in pain.		
	The prospective non-randomized pilot 9-month period study, *n* = 50 patients. After total knee arthroplasty (TKA), the solution contained a cocktail of ropivacaine, clonidine, epinephrine, and ketorolac with 1 mL of methylprednisolone (40 mg), which was infiltrated in the periarticular tissues, control group was second knee with the infiltration the same solutions mixture except for methylprednisolone. Patients’ follow-ups for pain evaluation were the first three days after surgery.	The reduction in pain with the periarticular infiltration ropivacaine and methylprednisolone cocktail was significant [44].
8. Botulinum toxin products. Product name and description: OnabotulinumtoxinA—the regulation of response to nociception in various diseases.		
	The multicenter, double-blind, randomized, placebo-controlled study enrolled 176 patients, and 158 completed the study. Randomization: i.a. onabotulinumtoxinA 400 U or 200 U and placebo (saline), followed up for 24 weeks.	Follow-up data revealed no significant differences between the treatment and placebo groups in the reduction in WOMAC mean pain score. Treatment was safe and without serious effects [45].
9. Combination nonsteroidal anti-inflammatory drug with HA. Product name and description: Diclofenac etalhyaluronate (DF) molecule covalently bonded to HA has a long-lasting anti-inflammatory effect.		
Phase 3	The multicenter, open-label, noncomparative study, *n* = 166 patients. Treatment regimen was i.a. 30 mg DF-HA every 4 weeks for 1 year (13 times in total). Follow-up 52 weeks.	The results showed that DF-HA was well tolerated and the data suggest a sustained improvement in the symptoms of OA [46].
Phase 3	Multicenter, randomized, double-blind, placebo-controlled trial, *n* = 440 patients. DF-HA 30 mg or placebo once every 4 weeks for 20 weeks (a total of 6 injections). Follow-up for 24 weeks.	The significant improvement in the WOMAC pain subscale score compared to placebo was over 12 weeks. The observed anaphylactic reactions will clarify the need for the safety of this drug [47].

## Data Availability

Publicly available datasets were analyzed in this study and are referred to in the list of references.

## Supplementary Materials

The following supporting information can be downloaded at: https://www.mdpi.com/article/10.3390/medicina60111819/s1, Table S1: Local treatments: advantages and disadvantages.
