# Supplementary material for: The Challenges of Local Intra-Articular Therapy
Source: Medicina (Kaunas). 2024 Nov 5;60(11):1819. doi: 10.3390/medicina60111819 (PMC11596802; doi:10.3390/medicina60111819)
Supplement: Supplementary file 1 [file medicina-60-01819-s001.zip › medicina-3262275-supplementary.pdf]

Table S1. Local treatments: advantages and disadvantages.

Local treatments	Target of action	Advantages	Disadvantages	Comments
Open synovectomy	Surgical removal of inflamed synovial tissues	Good access for removing synovia. Biomarkers testing in the post-operative synovium [1][2].	Pain and possible healing complications associated with the open surgery.	Large joints are usually chosen for this intervention.
Arthroscopic synovectomy	Minimally invasive surgical removal of the inflamed synovial tissues.	Quicker recovery, less post-operative pain, and faster rehabilitation [3] Biomarkers testing in the postoperative synovium [1][2].	Incomplete removal of the synovium due to technical reasons.	Technical advances in arthroscopy, including new microscopy techniques and video imaging, can be fully exploited for the prevention of joint damage. [3][4].
Intra-articular steroids injection	Anti-inflammatory effects	Low doses of steroids have a positive effect on cartilage structure and inhibit the number of inflammatory cells and the inflammation process. Low doses of steroids with prolonged release and slow action are recommended [5][6].	High doses of steroids have a detrimental effect on cartilage degradation. Each steroid injection must be administered judiciously to avoid cumulative effects and cartilage damage [6].	The EULAR recommendations for intra-articular therapy for different IAs should be taken into account [7].<br>The local acute synovitis suppression and pain relief response is rapid, effective, and lasts from two to six weeks. Currently, a low dose with a slow effect is recommended as a treatment strategy for synovitis in OA [5].
Radionuclide synovectomy	The basic principle of radiosynovectom y is to select radionuclides that emit high-	Radiosynovectomy (RVS) ablates the inflamed synovial membrane with then develops fibrosis. This technique can treat synovitis of various	Secondary radiosynovitis can occur two to four weeks after the procedure. In rare cases, a complication of infection is reported, and	The European Association of Nuclear Medicine (EANM) has approved guidelines for radionuclide synovectomy [9].<br>Three radionuclides are currently in use: Yttrium-90 (90Y-silicate/citrate), Rhenium-186 (186Re-sulfide), and Erbium-169 (169Er

	energy β-radiation. This radiation can therefore induce water hydrolysis with the production of reactive oxygen species and induce cell apoptosis due to oxidative stress.	aetiologies. Small joints can also be selected. Clinical data show efficacy with residual benefits lasting up to 12 months. These data lead to the conclusion that it is a safe alternative to surgical synovectomy [8].	sometimes aseptic necrosis can be caused by an error in the radionuclide injection technique.	citrate). All procedures must be carried out using ultrasound. The radiopharmaceutical dose shall be based on the size of the joint. Only a few countries follow the European Association of Nuclear Medicine's recommendations for radionuclide synovectomies in clinical practice.

References:

1. Soroosh, S.G.; Ghatfan, A.; Farbod, A.; Meftah, E. Synovial Biopsy for Establishing a Definite Diagnosis in Undifferentiated Chronic Knee Monoarthritis. *BMC Musculoskelet. Disord.* **2023**, *24*, 23, doi:10.1186/s12891-023-06138-x.
2. Orr, C.; Vieira-Sousa, E.; Boyle, D.L.; Buch, M.H.; Buckley, C.D.; Cañete, J.D.; Catrina, A.I.; Choy, E.H.S.; Emery, P.; Fearon, U.; et al. Synovial Tissue Research: A State-of-the-Art Review. *Nat. Rev. Rheumatol.* **2017**, *13*, 463–475, doi:10.1038/nrrheum.2017.115.
3. Ike, R.W.; Kalunian, K.C. Will Rheumatologists Ever Pick up the Arthroscope Again? *Int. J. Rheum. Dis.* **2021**, *24*, 1235–1246, doi:10.1111/1756-185X.14184.
4. Wu, J.-P.; Walton, M.; Wang, A.; Anderson, P.; Wang, T.; Kirk, T.B.; Zheng, M.H. The Development of Confocal Arthroscopy as Optical Histology for Rotator Cuff Tendinopathy. *J. Microsc.* **2015**, *259*, 269–275, doi:10.1111/jmi.12260.
5. Paik, J.; Duggan, S.T.; Keam, S.J. Triamcinolone Acetonide Extended-Release: A Review in Osteoarthritis Pain of the Knee. *Drugs* **2019**, *79*, 455–462, doi:10.1007/s40265-019-01083-3.
6. Wernecke, C.; Braun, H.J.; Dragoo, J.L. The Effect of Intra-Articular Corticosteroids on Articular Cartilage: A Systematic Review. *Orthop. J. Sports Med.* **2015**, *3*, 2325967115581163, doi:10.1177/2325967115581163.
7. Uson, J.; Rodriguez-García, S.C.; Castellanos-Moreira, R.; O'Neill, T.W.; Doherty, M.; Boesen, M.; Pandit, H.; Möller Parera, I.; Vardanyan, V.; Terslev, L.; et al. EULAR Recommendations for Intra-Articular Therapies. *Ann. Rheum. Dis.* **2021**, *80*, 1299–1305, doi:10.1136/annrheumdis-2021-220266.
8. Caballero Motta, L.R.; Anzola Alfaro, A.M.; Janta, I.; Molina Collada, J.; Henao, Y.K.; Pérez Pascual, R.; Álvaro-Gracia, J.M.; Nieto-González, J.C. Radiosynovectomy in Routine Care: An Old Tool with Modern Applications. *Ther. Adv. Musculoskelet. Dis.* **2021**, *13*, 1759720X211055309, doi:10.1177/1759720X211055309.

9. Kampen, W.U.; Boddenberg-Pätzold, B.; Fischer, M.; Gabriel, M.; Klett, R.; Konijnenberg, M.; Kresnik, E.; Lellouche, H.; Paycha, F.; Terslev, L.; et al. The EANM Guideline for Radiosynoviorthesis. *Eur. J. Nucl. Med. Mol. Imaging* **2022**, *49*, 681–708, doi:10.1007/s00259-021-05541-7.
